# Hypodermic Injections in Sciatica, Heart Disease, &c.

**Published:** 1862-05

**Authors:** S. Barrett


ART. III.—*Hypodermic Injections in Sciatica, Heart Disease, &c.; by* S. BARRETT, M. D.

[Communicated for Buffalo Medical and Surgical Journal.]

Mr. N., aged 60, gardener, was taken with severe sciatica of the left hip, extending down the outside of the leg to the foot, which rendered him unable to move the leg or foot without crying out with pain; the pain was so severe for three days and nights before I saw him, that it kept him in a constant state of perspiration.

With a hypodermic syringe I injected a solution of atropia and morphine $\frac{1}{8}$ f. ʒ into the cellular tissue under the trocantor major, on the evening of the 10th instant; the pain was relieved in ten minutes so that he could bend the knee and move the foot with very little pain; he had a refreshing sleep that night, and the next day resumed his labors as gardener; some stiffness and slight pain remaining in the course of the sciatic nerve. While on his way to work the second day he called at my office, and I injected a similar quantity a little lower down in the course of the nerve; since which time he has been free from pain or soreness.

In a case of organic disease of the heart of a man aged 72, near its close, he had not slept quietly for a week or more; I injected $\frac{1}{8}$ f. ʒ of the solution into the bend of the left arm at evening; it procured him a quiet night's rest and freedom from oppressed breathing for twenty-four hours, and by repeating it in twenty-four hours, it mitigated his sufferings beyond anything I have ever been able to do by any other means, in similar cases.

J. B., subject to severe paroxysms of asthma, which usually lasted one week or more; the second day of a severe attack I injected a solution of atropia and morphine into the bend of the left arm; it arrested the paroxism, and he slept all night.

I have found the combination of atrophia gr. ss., sulph. morphine gr, ii aqua pura, ʒj, prompt, and more permanent in its effects than either alone, and in many chronic and neuralgic diseases the most potent and useful hypodermic means of medication we possess.

